# A Simple Approach for COnsumption and RElease (CORE) Analysis of Metabolic Activity in Single Mammalian Embryos

**DOI:** 10.1371/journal.pone.0067834

**Published:** 2013-08-15

**Authors:** Fabrice Guerif, Paul McKeegan, Henry J. Leese, Roger G. Sturmey

**Affiliations:** 1 Centre for Cardiovascular and Metabolic Research, Hull York Medical School, University of Hull, Hull, United Kingdom; 2 Service de Médecine et Biologie de la Reproduction, CHRU de Tours, Tours, France; 3 Université François Rabelais, Tours, France; 4 INRA, UMR85 PRC, Nouzilly, France; Justus-Liebig-Universität, Germany

## Abstract

Non-invasive assay of the consumption and release of metabolites by individual human embryos could allow selection at the cleavage stage of development and facilitate Single Embryo Transfer in clinical IVF but will require simple, high throughput, sensitive methods applicable to small volume samples.

A rapid, simple, non-invasive method has therefore been devised using a standard fluorescence plate reader, and used to measure **t**he consumption of pyruvate and glucose, and release of lactate by single bovine embryos at all stages of preimplantation development in culture; amino acid profiles have been determined using HPLC.

Early embryos with an ‘intermediate’ level (6.14±0.27 pmol/embryo/h) of pyruvate uptake were associated with the highest rate (68.3%) of blastocyst development indicating that a mid “optimum” range of pyruvate consumption correlates with high viability in this bovine model.

## Introduction

There is a consensus move in clinical IVF towards single embryo transfer (SET) in order to reduce the incidence of multiple pregnancies. Elective SET is largely applied to blastocyst-stage embryos, since these have higher implantation rates than those at the cleavage stage [1]. However, despite the fact that embryo culture conditions *in vitro* support early development successfully, they are likely to be suboptimal compared to the *in vivo* environment [2] and there is evidence that prolonged culture may increase the risk of deleterious consequences [3] including alteration of gene expression, particularly of imprinted genes [4]–[6]. Identification of biomarkers of embryo viability early in development, which complement morphological evaluation, wouldt avoid the need to culture embryos until the blastocyst stage. One possible route to discovering such a biomarker of embryo viability lies in the study of early embryo metabolism, much of the stimulus for which has been driven by the notion that metabolic changes may be measured non-invasively and be predictive of viability.

Mammalian preimplantation embryo metabolism follows a widely accepted general pattern, which has been extensively reviewed [7]–[9]. During the cleavage stages, early embryos predominantly consume pyruvate, which is largely oxidised to CO_2_ [10] but, at least in the human, may also be converted to lactate [11]. Glucose depletion at this time is relatively low. At cavitation, glucose consumption increases markedly, giving an appearance of a “glycolytic phenotype” *in vitro*. However, the major source of ATP generation continues to be via oxidative phosphorylation, as demonstrated by a marked rise in O_2_ consumption at the blastocyst stage [12]–[14]. This general pattern appears to operate in most mammalian species studied [9]. In addition to glucose, lactate and pyruvate, amino acids may act as an energy source as well as playing a variety of other metabolic roles during preimplantation development [7]. A crucial discovery of recent years has been the realization that endogenous stores can also provide energy during early embryo development (for reviews, see: [15], [16].

A number of reports have proposed a link between metabolic activity and embryo viability; for example, those of Conaghan et al [17] for pyruvate, Houghton et al [14] for oxygen, Gardner and Leese [18] and Gardner et al [19] for glucose and Houghton et al [20] and Brison et al [21] for amino acids. However, the patterns that link single metabolite markers with viability are contested, with some reports proposing that low substrate consumption is indicative of high viability [22] while others suggest the converse [19]. Metabolomic approaches, which differ from studies of metabolism by screening the whole repertoire of small molecules, initially gave promising results for early embryo selection [23], [24]. However, the patterns that link metabolism and viability using this technology are not clear-cut and a recent prospective randomized trial has shown that the metabolomic approach did not improve the chance of IVF success and needed further development before it could be used as an objective marker of embryo viability [25]. Thus, there is a need for a simple, high throughput method to enable further investigations of embryo metabolism in a range of settings to build the knowledge set prior to possible translation of metabolic selection of embryos into a clinical setting. However progress toward this goal has been impeded by the need to use methods that are technically demanding and require specialist complex equipment in order to carry out metabolite assays on very small volume samples (5 µl–10 µl) with sufficient sensitivity to detect the metabolic activity of single preimplantation embryos.

In this paper, we have therefore refined and simplified the non-invasive enzymatic determination of spent embryo culture medium, based largely on the work of Leese and Barton [26], for use in a modern fluorescence plate reader. In vitro produced bovine embryos have been used as a model species. As well as describing the technique, we have measured the depletion of glucose and pyruvate, and the production of lactate on the same cohort of embryos in individual culture throughout preimplantation development and compared these results to established equivalent data as a means of validating the method. In addition, we have measured the depletion and appearance of a mixture of amino acids by HPLC to generate a comprehensive picture of metabolite consumption and release (CORE) by embryos in vitro. Using this approach, we have been able to relate metabolic activity to the ongoing developmental progress of single embryos.

## Materials and Methods

All chemicals were obtained from Sigma-Aldrich Chemical (Poole, UK) unless otherwise indicated. All enzymes for metabolic analysis were supplied by Roche (Burgess Hill, UK).

### In vitro production of bovine embryos

Ovaries were collected from a local abattoir and transported in Phosphate-Buffered Saline (PBS) at 39°C to the laboratory within 3 h of slaughter. Animal tissue was collected with full permission from slaughterhouse (ABP, Murton, York, UK). Upon arrival in the laboratory, the ovaries were washed twice with fresh warmed PBS and maintained at 39°C until aspirated.

Production of bovine embryos was performed as described previously [27]. Oocyte maturation was carried out in bicarbonate-buffered TCM-199 supplemented with 10% fetal bovine serum, 0.01 IU FSH/LH (Ferring Pharmaceutical, Langley, UK), 0.40 µg epidermal growth factor ml^−1^ and 2.2 ng fibroblast growth factor ml^−1^, in groups of 50 in 500 µl, incubated for 24 h at 39°C under humidified 5% CO_2_ in air.

Mature oocytes were fertilized with spermatozoa prepared from frozen-thawed semen samples from a single bull of proven fertility. Motile spermatozoa were collected after centrifugation on a discontinuous Percoll (Pharmacia Biotech, St Albans, UK) gradient (45∶90%) at 760×*g* for 30 min at room temperature. Spermatozoa and oocyte cumulus complexes (OCCs) were co-incubated in fertilization-TALP (Fert-TALP; [28]) for 18–24 h under the same conditions as described for oocyte maturation.

At 18–24 h post-insemination, putative zygotes were placed in groups of 20 in 20 µl droplets of a modified SOFaaBSA medium without lactate, under mineral oil and incubated at 39°C in a humidified atmosphere of 5% CO_2_, 5% O_2_ and 90% N_2_.

Determinations of the COnsumption of pyruvate and glucose, and RElease of lactate as well as amino acid COnsumption and Release (CORE analysis) were performed on two- to four-cell, eight-cell embryos, morula, early blastocysts, expanded blastocysts and hatched blastocysts stages (days 2, 3, 5, 6, 7 or 8 of culture, respectively). Morphological assessment of blastocysts was based on the expansion of the blastocoele cavity (B1 to B7) [29] B1–B2 stages were defined as early blastocysts, B3–B4 stages were defined as expanded blastocysts, whereas B5–B7 stages were defined as hatching/hatched blastocysts.

### Individual culture of embryos for metabolic analysis

Embryos were moved from SOFaaBSA culture medium to SOFaaBSA “analysis” medium, which contained 0.5 mmol glucose l^−1^ and no lactate, but was identical to SOFaaBSA in all other respects. After two washes in SOFaaBSA “analysis” medium, embryos were incubated individually for 24 h in 4 µl droplets of this medium under mineral oil at 39°C in a humidified atmosphere of 5% CO_2_, 5% O_2_, 90% N_2_. Control droplets of medium, incubated adjacent to the droplets containing the embryos, were used as controls to account for non-specific metabolite degradation/appearance. Samples were frozen under oil at −80°C until analysis.

After 24 hours of culture, the morphological status of each embryo was recorded and compared to the stage recorded at the beginning of individual culture. Embryos with morphological progression were defined as follows: increase in the number of cells for cleaved embryos and progression towards a higher stage for morula, early and expanded blastocyst (ie: morula to B1 for morula, B1 to B2 for early blastocysts and B4 to B5 for expanded blastocysts). By analyzing the difference between substrate levels in incubation droplets and controls, measurements could be made on individual embryos. In this way, a ‘CORE’ profile of individual bovine embryo metabolism from day 2 to day 8 was produced.

### Determination of pyruvate and glucose uptake and lactate production

Analysis of the control and embryo samples of media was performed with a Tecan® Infinite M200 spectrophotometer using an ultramicrofluorometric technique adapted from those of Leese and Barton (26); Gardner and Leese [30] and Leese, [31]. This modified method uses a 96-well plate system and enables the rapid measurement of depletion or appearance of metabolites by a number of individual embryos in parallel in a high throughput manner. The assay mixtures (details below) were excited at 340 nm with emitted fluorescence measured at 460 nm to give a background fluorescent value. Sample/control medium (1 µl) was added to 10 µl of assay mixture and fluorescence recorded as described above.. In this way, changes in fluorescence due to NADH oxidation or NAD^+^/NADP^+^ reduction were recorded and calibrated against a series of standards. All reagents and samples were kept on ice, and reactions carried out at 25°C. Uptake or production over the 24 h period were determined in triplicate against a freshly prepared six point standard curve for each compound and expressed as pmol/embryo/h. Results are expressed as means ± SEM. For each measure, the confirmed variance of assay was <5%.

### Assays for pyruvate

Sample medium (1 µl) was added to 10 µl of assay mixture containing 0.1 mM NADH and 40 IU lactate dehydogenase ml^−1^ in 4.6 mM EPPS buffer, pH 8.0, and incubated at 25°C for 3 minutes. Changes in fluorescence due to NADH oxidation were monitored. Final concentrations in each spent culture droplet were determined against a six point standard curve from 0–0.45 mM pyruvate.

### Assays for glucose

Sample medium (1 µl) was added to 10 µl of assay mixture containing 0.4 mM dithiothreitol, 3.07 mM MgSO_4_, 0.42 mM ATP, 1.25 mM NADP^+^, 20 IU hexokinase/glucose-6-phosphate dehydrogenase (HK/G6PDH) ml^−1^ in EPPS buffer at pH 8.0, and incubated at 25°C for 10 minutes. Changes in fluorescence due to NADP^+^ reduction were monitored. Final concentrations in each spent culture droplet were determined against a six point standard curve from 0–0.5 mM glucose.

### Assays for lactate

Sample medium (1 µl) was added to 10 µl of assay mixture containing 40 IU lactate dehydrogenase (LDH) ml^−1^ in a glycine-hydrazine buffer, pH 9.4, and incubated at 25°C for 30 minutes. Changes in fluorescence due to NAD^+^ reduction were monitored. Final concentrations in each spent culture droplet were determined against a six point standard curve from 0–1.0 mM lactate.

### Determination of amino acid profiles

Spent and control droplets were diluted 1∶12.5 in high performance liquid chromatography (HPLC) grade water. Amino acid concentrations in the droplets were determined by reverse-phase HPLC as described previously [20], [32]. Analysis was performed on an Agilent 1100 automated HPLC system fitted with a Phenomenex Hyperclone 5 µM C18 ODS 250×4.6 mm column (Phenomenex, Macclesfield, UK). A net fall in amino acid concentration in the spent culture droplet was interpreted as “consumption”; a net increase of an amino acid as “release”. The sum of consumption and production was used as an overall indicator of amino acid metabolic activity and termed “turnover”.

### Prospective selection of viable embryos as a function of pyruvate uptake on day 2

A further experiment focusing on embryo pyruvate consumption on day 2 was carried out. On day 2, thirty embryos were cultured individually for 24 hours for metabolic analysis. Pyruvate uptake for each embryo was measured, and on day 3, the 30 embryos were allocated into tertiles on the basis of their pyruvate consumption. Group 1 comprised 10 embryos with the lowest measured values of pyruvate depletion; Group 2 comprised 10 embryos with intermediate pyruvate uptake whereas embryos assigned to Group 3 had the 10 embryos with the highest pyruvate uptake. This approach was chosen to avoid bias by ensuring that final group sizes were all equal. The 3 groups of 10 embryos were cultured from day 3 to day 8 and the rate of blastocyst development on day 8 was recorded for each group. This experiment was repeated 6 times.

### Experimental design and Statistical analysis

Glucose and pyruvate consumption, production of lactate and amino acid consumption/production/turnover by embryos are expressed as pmol per embryo per hour ± SEM. Embryos were assayed at one developmental stage only in order to minimize the stress of single culture. In the prospective studies, pyruvate alone was measured in triplicate to provide additional confidence in the results. Significant differences in metabolic profile (glucose, lactate and pyruvate) were tested by Student's t-test and one-way analysis of variance (ANOVA) followed by Fisher's LSD test *post-hoc* using Statview® (version 5.1). Following normality tests, amino acid data were tested by Kruskal-Wallis tests followed by Mann-Whitney-U tests *post hoc* since they were not normally distributed.

## Results

### Carbohydrate consumption/release throughout development

Pyruvate and glucose uptake and lactate production were recorded from individual *in vitro*-produced bovine embryos throughout preimplantation development. An overall increase in pyruvate and glucose uptake with development was observed (**Table 1**). Pyruvate uptake was relatively constant up until the 5–8 cell-stage then increased progressively until the expanded blastocyst-stage. This rise was followed by a decrease at the time of hatching to the level observed for early blastocysts. Glucose consumption followed a broadly similar pattern with relatively low consumption until the early blastocyst-stage. By contrast to pyruvate, glucose uptake increased dramatically from early to hatched blastocysts. Lactate production followed a similar pattern to the uptake of glucose with a dramatic increase from the morula stage.

**Table 1:** The uptake of pyruvate and glucose and the production of lactate by individual bovine embryos produced *in vitro*.

Day of culture	Stage	Pyruvate uptake(pmol/embryo/h)	Glucose uptake(pmol/embryo/h)	Lactate production(pmol/embryo/h)
1–2	2–4 cells	5.08±0.44^a,b,c,d^ (88)	2.33±0.26^a,b,c,d^ (83)	1.67±0.24^a,b,c,d^ (118)
2–3	5–8 cells	5.43±0.48^e,f,g,h^ (74)	2.59±0.37^e,f,g^ (56)	3.62±0.42^e,f,g,h^ (106)
4–5	Morula	9.51±0.89^a,e,i,j,k,l^ (31)	7.20±1.28^a,h,i,j^ (32)	14.72±1.20^a,e,i,j,k^ (44)
5–6	Early blastocysts	16.43±1.26^b,f,i,j,m^ (54)	14.31±1.14^b,e,h,k,l^ (57)	23.62±1.40^b,f,i,l,m^ (74)
6–7	Expanded blastocysts	22.03±1.02^c,g,k,m,n^ (47)	34.23±1.82^c,f,i,k,m^ (85)	51.12±2.39^c,g,j,l,n^ (123)
7–8	Hatched blastocysts	16.76±2.11^d,h,l,n^ (26)	56.29±2.91^d,g,j,l,m^ (31)	85.98±4.47^d,h,k,m,n^ (46)

Number of samples are indicated in brackets.

Values with the same superscript in each column are significantly different (p<0.05).

### Amino acid profile throughout development

**Figure 1:**
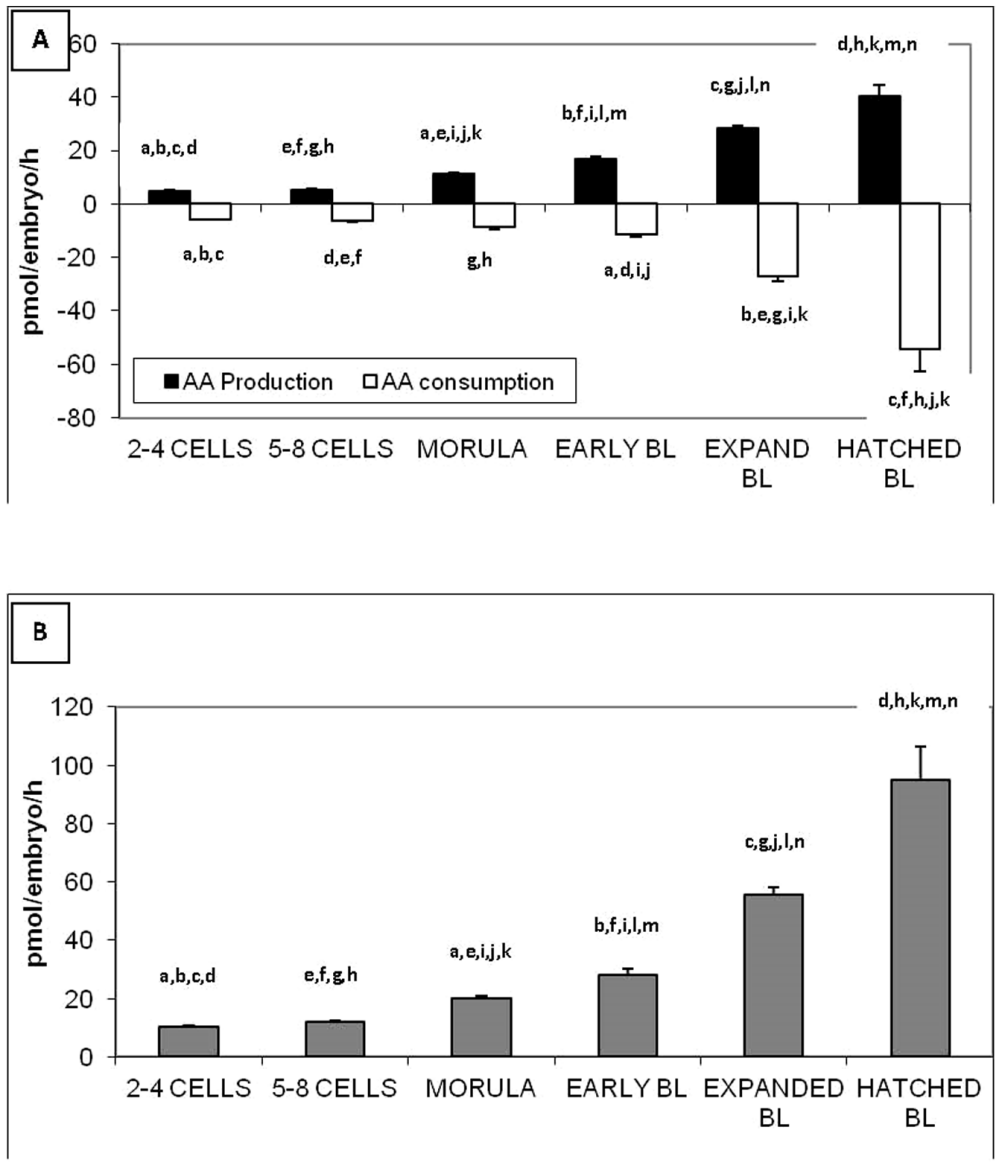
Amino acid production/consumption (A) and turnover (B) throughout preimplantation development by individual bovine embryos produced *in vitro*. Turnover is the sum of amino acid consumption and production. Values with the same superscript are significantly different (p<0.05).

The overall release of AA increased with development, from 4.78±0.23 pmol/embryo/h for the 2–4 cell-stage to 40.26±4.14 pmol/embryo/h for hatched blastocysts (p<0.05) (**Fig. 1A**). Similarly, the overall consumption of AA increased with development from −5.72±0.35 pmol/embryo/h for 2–4 cell-stage to −54.57±7.71 pmol/embryo/h for hatched blastocysts (p<0.05) (**Fig. 1A**). As a consequence, the turnover, which is the sum of production and consumption of amino acid also increased with development from 10.49±0.39 pmol/embryo/h for 2–4 cell-stage to 94.84±11.64 for hatched blastocysts (p<0.05 (**Fig. 1B**). The data from which these figures are drawn is included in **Table S1**.

### Metabolic analysis and morphological progression after 24 hours of individual culture

There was no difference in glucose depletion between cleavage-stage embryos that progressed and those that did not during a 24 hr culture period. However, early and expanded blastocysts that showed morphological changes after 24 hours of culture consumed significantly higher levels of glucose (p = 0.0023 and p<0.0001, respectively, **Table 2**). By contrast, differences in lactate production between embryos with morphological progression were observed from the morula stage. Pyruvate uptake was significantly higher for embryos with morphological progression at all the stages of development measured in the present study. In particular, 2–4 cell-stage embryos that progressed morphologically had significantly higher pyruvate uptake than those which did not progress (6.19±0.66 vs. 3.80±0.52 pmol/embryo/h, respectively, p<0.05, **Table 2**)

**Table 2:** Comparison of mean values of metabolites consumed or produced in culture media between embryos with or without evidences of morphological changes after 24 hours of individual culture.

Metabolite	Initial stage	No morphological changes	Morphological changes	*p*
Pyruvate	2–4 cells	3.80±0.52 (41)	6.19±0.66 (47)	0.0062
uptake	5–8 cells	4.26±0.54 (42)	6.97±0.80 (32)	0.0048
(pmol/emb/h)	Morula	6.99±0.98 (17)	12.57±1.15 (14)	0.0009
	Early blastocysts	11.53±1.47 (19)	19.09±1.61 (35)	0.0031
	Expanded blastocysts	19.26±1.26 (22)	24.47±1.41 (25)	0.0092
Glucose	2–4 cells	2.76±0.44 (35)	2.38±0.49 (28)	0.5660
uptake	5–8 cells	2.62±0.61 (23)	2.52±0.52 (18)	0.9035
(pmol/emb/h)	Morula	6.29±1.70 (20)	8.71±1.93 (12)	0.3703
	Early blastocysts	9.54±1.36 (19)	16.70±1.43 (38)	0.0023
	Expanded blastocysts	25.60±2.05 (37)	40.88±2.42 (48)	<0.0001
Lactate	2–4 cells	1.24±0.32 (50)	0.94±0.22 (47)	0.4514
production	5–8 cells	4.36±0.70 (39)	3.60±0.67 (50)	0.4419
(pmol/emb/h)	Morula	12.28±1.53 (26)	18.24±1.64 (18)	0.0128
	Early blastocysts	16.81±1.40 (24)	26.90±1.80 (50)	0.0005
	Expanded blastocysts	38.21±3.00 (48)	59.39±3.06 (75)	<0.0001
Amino acid	2–4 cells	10.38±0.57 (25)	10.62±0.55 (23)	0.7630
turnover	5–8 cells	12.71±0.69 (21)	11.50±0.64 (31)	0.2152
(pmol/emb/h)	Morula	17.25±0.92 (30)	24.67±1.44 (18)	<0.0001
	Early blastocysts	23.19±1.34 (29)	35.29±3.21 (21)	0.0003
	Expanded blastocysts	47.50±2.64 (36)	64.28±3.71 (34)	0.0004

Number of samples analysed are indicated in brackets.

Amino acid turnover is the sum of consumption and production.

**Figure 2:**
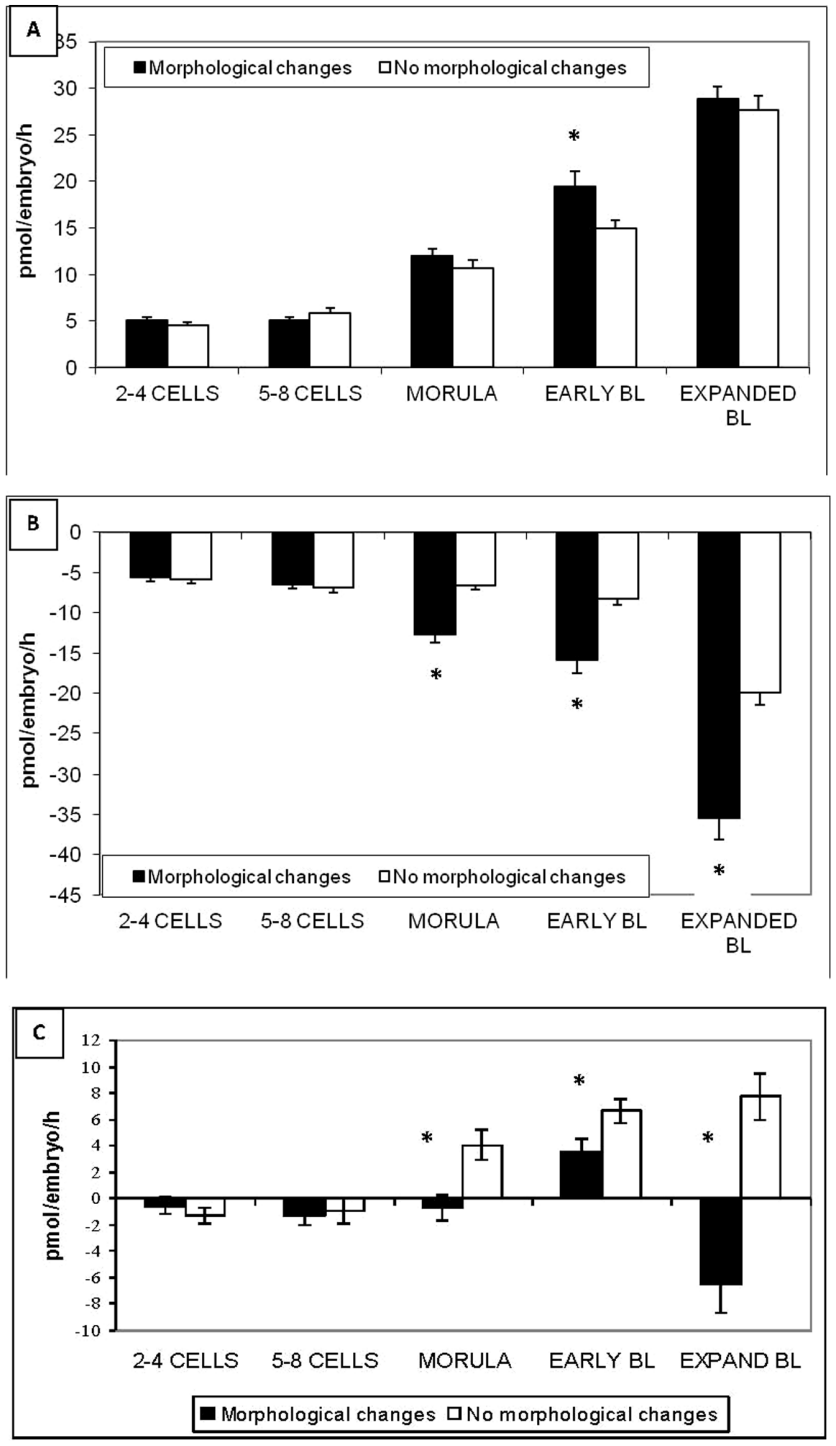
Amino acid production (A) and consumption (B) and balance (C) throughout preimplantation development by individual bovine embryos produced *in vitro.* Comparison between embryos with or without morphological changes after 24 hours of individual culture. Balance is the difference between amino acid consumption and production. * = p<0.05.

The turnover of amino acids, which can be used as an overall marker of amino acid metabolic activity was similar between embryos with and without morphological changes at the early stages within a 24 hr period (2–4 cells and 5–8 cells) (**Table 2**). However, the turnover at later stages (morula, early and expanded blastocysts) was significantly higher for embryos that subsequently exhibited morphological changes in comparison with embryos without morphological changes after 24 hours of culture. This observation was mainly explained by an increase in amino acid consumption at these stages for embryos with morphological changes (**Fig. 2A, 2B and 2C**). The data from which these figures are drawn are included in **Table S2.**

### Pyruvate consumption between day 2 and day 3 as a prospective marker of ongoing development

**Figure 3:**
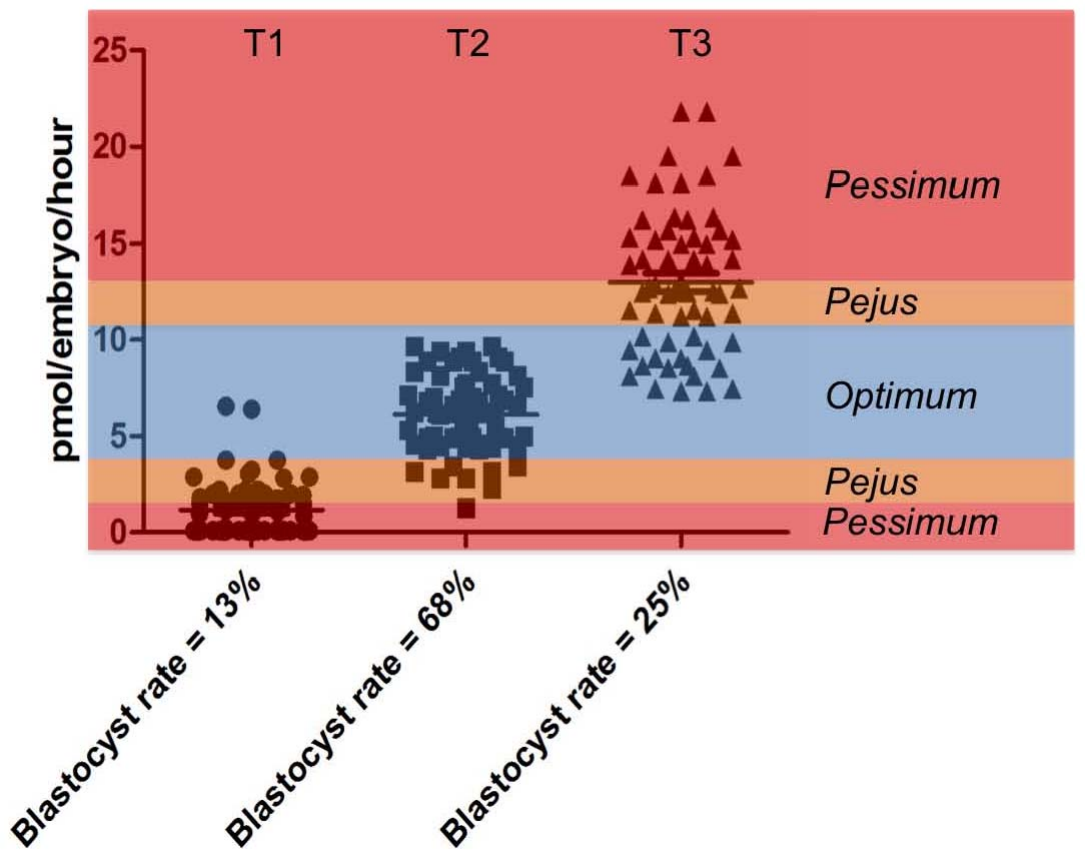
Individual values for pyruvate consumption by bovine embryos assigned to tertiles on the basis of their pyruvate consumption and the resultant blastocyst rates. Tertile 1 (T1) are the cohort of embryos with low rates of pyruvate consumption (1.14±0.02 pmol/embryo/h); Tertile 2 (T2) are the embryos with an “intermediate” (6.14±0.27 pmol/embryo/h) level of pyruvate consumption and Tertile 3 are the embryos with high pyruvate consumption (13.00±0.48 pmol/embryo/h). Categories of activity, according to the Dynamic Energy Budget Theory are overlaid. According to this theory, we propose that for any given substrate, there is an optimum level of metabolism, and that this optimum varies temporally to meet the demands of growth and development at each stage. In the case of an early embryo, we propose that when exposed to a challenge or stress, the resulting response will have an energy cost. This cost may be met by an increase depletion of a given substrate to fuel the additional activity, or indeed may lead to a partitioning of substrates away from essential processes. In either case, a shift out of the optimum range leads to the early embryo having a *pejus* metabolism, indicating sub-viability. The early embryo in this stage of metabolism may be able to rectify the stress and return to optimum metabolism and continue development, however if further resources are needed, the embryo may be further compromised as indicated by a *pessimism* metabolic phenotype; in this case, very low or very high metabolic activity indicative of sub viability.

Given that pyruvate depletion differed between embryos that progressed morphologically after 24 hours of individual culture we focused, in 6 independent experiments, on the relationship between pyruvate consumption of day 2 cleaving embryos and their ability to form a blastocyst on day 8. In each experiment, thirty day 2 embryos were cultured individually for 24 hours from day 2 to day 3. Then on day 3, embryos were grouped according to the level of pyruvate uptake measured after 24 hours of individual culture, and returned to culture until day 8. The thirty embryos were divided into 3 groups of ten embryos (low, medium and high level of consumption). The embryos with intermediate level of pyruvate uptake were associated with the highest number of cells on day 2 (5.43±0.16) and on day 3 (6.37±0.20) (**Table 3** **,** **Fig. 3**). The intermediate group was also associated with the highest rate of blastocyst development on day 8 in comparison with the groups that comprised embryos with low and high level of pyruvate uptake (68.3% vs 13.3% and 25.0%, respectively, p<0.05).

**Table 3:** Rate of blastocyst development according to the level of pyruvate uptake measured between day 2 and day 3.

	LOWpyruvate uptake	INTERMEDIATEpyruvate uptake	HIGHPyruvate uptake
n	60	60	60
Mean pyruvate uptake(pmol/emb/h)	1.14±0.20^a^	6.14±0.27^a^	13.00±0.48^a^
Nb cells (Day 2)	4.48±0.18^a^	5.43±0.16^a,b^	4.80±0.18^b^
Nb cells (Day 3)	5.15±0.21^a^	6.37±0.20^a^	5.80±0.17^a^
Blastocyst rate (Day 8)	8/60	41/60	15/60
	13.3%^a^	68.33%^a,b^	25.0%^b^

Values with the same superscript in each row are significantly different (p<0.05).

Data from 6 independent experiments including 30 embryos for each.

All stages (early, expanded and hatched) have been included to define the blastocyst rate.

## Discussion

In this study, we report a detailed analysis of individual bovine embryo metabolism, measuring a number of metabolic markers to provide a comprehensive picture of substrate utilization *in vitro*. We have generated these data by modifying the ultramicrofluorometric method of Leese and Barton [26] to produce a high throughput, simple spectrophotometric technique that could be used in any laboratory with access to a plate reader with fluorescence detection. Previously, only one study focused on the analysis of the uptake of pyruvate and glucose and the production of lactate by bovine embryos produced *in vitro* and incubated in groups [33]. We chose this key data as the standard against which to compare the data for individual embryos generated by our modified, plate-reader based enzymatic assays. The results from the present study broadly agree with those reported by Thompson et al [33], suggesting that our refinement of the technique generates robust data. However, in the present study, blastocysts have been sub-categorised according to blastocoel development to give a more detailed picture of metabolic activity throughout *in vitro* development. Our data confirm that in bovine embryos, pyruvate is consumed in preference to glucose in the early stages of development before falling at the hatched blastocyst stage. Glucose becomes the predominantly depleted substrate at the blastocyst stage with a dramatic increase both in glucose uptake and lactate production, during expansion and hatching. In our study, the highest value of glucose uptake was observed for hatched blastocysts (56 pmol/embryo/h); a value similar to that described for human blastocysts (42 pmol/embryo/h) [34]. Similarly, the rate of lactate production during hatching (86 pmol/embryo/h) was comparable to the highest value observed on day 5.5 for human blastocysts (95 pmol/embryo/h) [35]. It was notable that the mean glucose uptake throughout development, and the mean lactate production from the morula stage were approximately 2-fold higher in our study compared to values reported by Thompson et al [33]. Our results were obtained using a lactate-free culture medium, whereas in the study of Thompson et al [33] lactate was present at a concentration of 10 mM which may have inhibited the conversion of glucose to lactate by glycolysis, as suggested by Leese [36]. The high levels of lactate formed in the later stages of development could be in preparation for the anoxic environment that the embryo may encounter at implantation [18], [37], [38].

During blastocyst expansion, more glucose was depleted than could be accounted for by lactate appearance, suggesting that glucose may be diverted away from aerobic glycolysis. The fate of this glucose is unclear, however it is likely that it is either oxidized completely via the TCA cycle or utilized in one of the other glucose-consuming pathways such as the Pentose Phosphate Pathway to provide biosynthetic precursors. This mirrors the pattern seen in porcine embryos [39], but differs slightly from the picture for human embryo metabolism [35].

In addition, the current study has measured the metabolism of a mixture amino acids, again collecting data from single embryos representing a variety of preimplantation developmental stages. Amino acid metabolism, as indicated by both consumption and release, increased throughout development especially during blastocyst expansion and hatching. Somewhat surprisingly, there was a net appearance of amino acids during blastocoel formation whereas hatching was associated with a net depletion. There were no differences in overall usage of individual amino acids between early stage embryos (2–4c, 5–8c and morula) that developed compared to those which did not develop during the 24 h assay culture period. This is in agreement with our earlier work [40] which found that amino acid metabolism was only predictive of bovine embryo development at the zygote stage. A number of differences were apparent at the blastocyst stage: for example, early blastocysts that developed during the assay period consumed more arginine and produced less leucine than those that did not develop during the culture period. The observed differences in amino acid metabolism by blastocysts that developed might provide additive value in embryo selections if combined with pyruvate consumption during cleavage (see below). Experiments to test this proposition are ongoing.

It was of interest that the overall level of amino acid metabolism at the blastocyst stage was slightly elevated compared to our previous work on the bovine [40], although values for early stage embryos in the two studies were broadly comparable. Differences could be attributable to individual embryo variation, however it is notable that the semen used in this study was from a different sire to that in our previous work [40]. This raises the interesting question of the degree to which embryo metabolism is influenced by the sperm used for in vitro fertilisation. There are of course a variety of other, if less likely, explanations for these differences, including changes in protocols, illustrating that care should be exercised when extrapolating data from different studies.

We feel that the novelty of the current work arises from determining metabolic activity of individually cultured embryos by a rapid process, minimizing the time that the embryos are cultured singly. This has provided the opportunity to track ongoing development and search retrospectively for links between developmental outcome and metabolic activity in the context of a range of identifiable substrates. It is important to highlight that bovine embryo in vitro development is compromised in single culture systems. Of all the substrates measured (glucose, pyruvate, lactate and 18 amino acids), the consumption of pyruvate differed most strikingly and consistently between embryos that progressed morphologically and those without morphological progression. Differences in pyruvate depletion were consistently observed at all stages examined, with embryos that completed a developmental progression consuming more pyruvate than those that remained developmentally static. This observation prompted us to ask whether the pattern in which cleavage stage embryos depleted pyruvate could predict the ability to generate a blastocyst.

In order to do this, embryos were divided prospectively into three groups on the basis of whether they had “high” (13.00±0.48 pmol/embryo/h) “Intermediate” (6.14±0.27 pmol/embryo/h) or “low” (1.14 pmol/embryo/h) pyruvate depletion on day 2 of development. Using this approach, it was found that embryos on day 2 of development with an ‘intermediate level’ of pyruvate uptake had a 68% probability of reaching the blastocyst stage in comparison with early embryos with a ‘low’ or ‘high’ level of pyruvate uptake (13% and 25% respectively). In related work, Conaghan *et al.*, [17] showed that pyruvate uptake by individual human embryos measured each 24 hours between day 1 and day 3 was significantly lower for embryos that implanted than for those which failed to implant. Our data suggest that, at least for pyruvate consumption, there is a middle ‘optimum’ range of depletion, which correlates with high viability. By contrast, embryos with elevated or reduced metabolism are developmentally compromised. This conclusion is consistent with the data of Turner et al [41] for pyruvate consumption by single human embryos conceived by natural cycle IVF.

These data suggest that embryo metabolic activity relates to ongoing developmental capacity and offer an opportunity to revisit the notion of a unifying framework to describe the relationship between metabolic activity and early development. One such framework is offered by the “Quiet Embryo Hypothesis” [22]. In summary, the Quiet Embryo Hypothesis, states that embryo viability is best served by a low metabolic activity that falls within a low range that can be termed ‘quiet’; the corollary states that an ‘active’ or ‘noisy’ metabolism is indicative of embryos requiring higher nutrient inputs and consequently having to expend extra energy, most likely required to carry out repair processes to reach the same developmental endpoint. This idea was developed by Leese et al [42] who proposed that the ‘concept of quiet metabolism is not about ’one size fits all’ but rather that, for any given set of circumstances there is an optimal range of embryonic activity consistent with successful developmental progression. The current data support this idea that there is an ‘optimum range’ of metabolic activity that relates to a minimal input to carry out developmental milestone events; below this threshold, the embryo will have insufficient metabolic activity to support development while activity of a given metabolic pathway above a given threshold may be indicative of a stressed embryo that is ‘working harder;’ to maintain its developmental trajectory. This notion is well-accommodated in the Dynamic Energy Budget scheme of Kooijman [43], which is a universal ecological concept that describes metabolism sufficient to meet the needs for maintenance and growth at any given stage of development in terms of an “optimum range”, and of ranges higher and lower than optimum, which, in the nomenclature of the Dynamic Energy Budget Theory, are referred to as ‘pejus’ and ‘pessimum’ ranges. In a state of “*pejus*”, there is still scope for demands to be met, but at a cost to growth/development, due to an increased metabolic burden required to respond to a stress. In the case of the early embryo, such a response could include early activation of the genome, repair of cellular damage, degradation of damaged blastomeres, amongst other possible processes; each will lead to a rise in energy metabolism and/or a potential partitioning of substrates away from other essential homeostatic processes, or a reduced ability to supply nutrients (i.e. insufficient metabolism). Alternatively, if an embryo is severely compromised, the “*pejus*” metabolism might be low, and sufficient for one or two cleavages, possibly reliant on endogenous energy reserves, but below the threshold required to sustain development to a blastocyst and beyond. Metabolic activity below or above the optimum therefore places the embryo in a compromised metabolic state (**Fig. 3**). Metabolic activity is necessarily dynamic and may not be fixed. As a consequence, in the case of a stressed embryo with a compromised metabolism, if the stress is rectified, the embryo may escape from the “*pejus*” range and return to the optimum range to continue its development. In other individual cases, the cognate increase in metabolic energy to meet rising demand will lead to an increase in substrate depletion, increased overall metabolic activity, and the appearance of a *hyperactive metabolism* leading to a state of *pessimism* (from the Latin for “the worst”). Examples of this might include a loss of metabolic regulation and subsequent degeneration most likely arising from damage to mitochondria or loss of REDOX regulation; the consequence is a drop to insufficient metabolic activity and ultimate degeneration.

In conclusion, we propose that the idea of a dynamic energy budget with an optimal range of metabolic activity provides an attractive evolution of the Quiet Embryo Hypothesis. The common thread is that metabolic homeostasis and restraint are conducive to development, and an embryo developing with a high degree of fidelity and low requirement for additional resources will be a low-input system with a metabolism that is optimised to meet requirements. We feel that our present data, combined with the findings of Conaghan et al [17] and Turner et al [41] which proposed a link between pyruvate metabolism and human embryo viability, and a reanalysis of the relationship between metabolism and viability make a strong case for revisiting pyruvate depletion as a biomarker of embryo viability, particularly given the availability of a rapid, high-throughput simple technique for the assay of single-substrate consumption.

## Supporting Information

Table S1 **Comparison of mean values of AA consumed or produced in culture media by individual bovine embryos produced** ***in vitro*** **.**
(DOC)

Table S2 **Comparison of mean values of AA consumed or produced in culture media between embryos with or without evidences of morphological changes after 24 hours of individual culture.**
(DOC)
