# Supplementary material for: A Simple Approach for COnsumption and RElease (CORE) Analysis of Metabolic Activity in Single Mammalian Embryos
Source: PLoS One. 2013 Aug 15;8(8):e67834. doi: 10.1371/journal.pone.0067834 (PMC3744531; doi:10.1371/journal.pone.0067834)
Supplement: Table S1 — Comparison of mean values of AA consumed or produced in culture media by individual bovine embryos produced in vitro . (DOC) [file pone.0067834.s001.doc]

**Table S1**

**Comparison of mean values of AA consumed or produced in culture media by individual bovine embryos produced *in vitro***

|  | ASP | GLU | ASN | SER | HIS | GLN | GLY | THR | ARG | ALA | TYR | TRP | MET | VAL | PHE | ISO | LEU | LYS |
| --- | --- | --- | --- | --- | --- | --- | --- | --- | --- | --- | --- | --- | --- | --- | --- | --- | --- | --- |
| 2-4 cells | 0.33  +0.05 | 0.28  +0.08 | -0.13  +0.03 | -0.06  +0.04 | -0.09  +0.04 | -2.41  +0.16 | 0.05  +0.03 | -0.06  +0.03 | -2.00  +0.22 | 2.26  +0.14 | 0.04  +0.03 | 0.05  +0.03 | 0.05  +0.01 | 0.13  +0.02 | 0.14  +0.03 | 0.08  +0.03 | -0.12  +0.04 | 0.51  +0.04 |
| 5-8 cells | 0.31  +0.05 | 0.38  +0.08 | -0.13  +0.03 | -0.03  +0.06 | -0.01  +0.06 | -1.70  +0.14 | 0.44  +0.1 | -0.06  +0.04 | -3.8  +0.33 | 1.83  +0.14 | 0.07  +0.01 | 0.24  +0.05 | 0.04  +0.01 | 0.16  +0.02 | 0.19  +0.03 | 0.09  +0.02 | -0.15  +0.05 | 0.92  +0.11 |
| Morula | -0.17  +0.15 | -1.55  +0.43 | -0.13  +0.07 | -0.65  +0.09 | 0.17  +0.09 | -0.24  +0.18 | 0.76  +0.08 | 0.08  +0.13 | -4.32  +0.25 | 5.74  +0.42 | 0.21  +0.03 | -0.02  +0.17 | 0.02  +0.01 | 0.3  +0.04 | 0.32  +0.03 | 0.13  +0.04 | 0.18  +0.05 | 1.45  +0.13 |
| Early Blasto | 038  +0.42 | -5.64  +0.54 | 0.02  +0.04 | -0.34  +0.11 | -0.03  +0.06 | 3.02  +0.39 | 0.51  +0.06 | 0.01  +0.04 | -3.49  +0.31 | 8.79  +0.56 | 0.22  +0.02 | 0.60  +0.06 | -0.02  +0.02 | 0.34  +0.05 | 0.37  +0.05 | -0.13  +0.05 | 0.26  +0.08 | 0.47  +0.12 |
| Expanded Blasto | -1.19  +0.59 | -12.45  +0.95 | -0.04  +0.05 | -0.54  +0.12 | -0.04  +0.05 | 5.47  +0.34 | 0.93  +0.23 | -0.01  +0.05 | -9.36  +0.64 | 16.00  +0.72 | 0.13  +0.03 | 0.87  +0.07 | -0.26  +0.03 | 0.25  +0.08 | 0.81  +0.11 | -0.6  +0.06 | -0.19  +0.12 | 0.99  +0.13 |
| Hatched Blasto | -6.09  +2.42 | -24.1  +2.83 | -0.04  +0.1 | -1.34  +0.46 | -0.04  +0.09 | 8.07  +1.49 | 0.87  +0.11 | -0.14  +0.09 | -17.91  +2.34 | 26.62  +2.87 | 0.11  +0.1 | 1.01  +0.21 | -0.67  +0.1 | -0.01  +0.17 | 1.26  +0.39 | -1.35  +0.24 | -1.49  +0.37 | 0.92  +0.3 |

Value are pmol/embryo/h and expressed as mean + SEM. For number of observations, please see main manuscript.

Negative values indicate amino acid consumption. Positive values indicate amino acid production.
