# Supplementary material for: A Simple Approach for COnsumption and RElease (CORE) Analysis of Metabolic Activity in Single Mammalian Embryos
Source: PLoS One. 2013 Aug 15;8(8):e67834. doi: 10.1371/journal.pone.0067834 (PMC3744531; doi:10.1371/journal.pone.0067834)
Supplement: Table S2 — Comparison of mean values of AA consumed or produced in culture media between embryos with or without evidences of morphological changes after 24 hours of individual culture. (DOC) [file pone.0067834.s002.doc]

**Table S2**

**Comparison of mean values of AA consumed or produced in culture media between embryos with or without evidences of morphological changes after 24 hours of individual culture.**

|  | ASP | GLU | ASN | SER | HIS | GLN | GLY | THR | ARG | ALA | TYR | TRP | MET | VAL | PHE | ISO | LEU | LYS |
| --- | --- | --- | --- | --- | --- | --- | --- | --- | --- | --- | --- | --- | --- | --- | --- | --- | --- | --- |
| 2-4 cells  NO MC | 0.34  +0.07 | 0.22  +0.1 | -0.14  +0.05 | -0.01  +0.06 | -0.1  +0.05 | -2.35  +0.22 | 0.08  +0.04 | -0.05  +0.04 | -2.1  +0.22 | 2.13  +0.18 | 0.05  +0.05 | 0.05  +0.03 | 0.03  +0.01 | 0.12  +0.03 | 0.12  +0.05 | 0.04  +0.04 | -0.17  +0.06 | 0.65  +0.17 |
| 2-4 cells  MC | 0.33  +0.05 | 0.33  +0.13 | -0.12  +0.03 | -0.11  +0.05 | -0.08  +0.05 | -2.47  +0.23 | 0.01  +0.04 | -0.08  +0.04 | -1.91  +0.39 | 2.4  +0.22 | 0.03  +0.03 | 0.05  +0.05 | 0.06  +0.01 | 0.14  +0.03 | 0.17  +0.03 | 0.13  +0.04 | -0.08  +0.05 | 0.39  +0.21 |
|  |  |  |  |  |  |  |  |  |  |  |  |  |  |  |  |  |  |  |
| 5-8 cells  NO MC | 0.36  +0.09 | 0.27  +0.12 | -0.2 ^g^  +0.05 | 0.07  +0.1 | 0.08  +0.08 | -1.79  +0.21 | 0.57  +0.15 | -0.03  +0.06 | -3.65  +0.42 | 1.65  +0.18 | 0.07  +0.02 | 0.22  +0.08 | 0.04  +0.02 | 0.13  +0.02 | 0.15  +0.05 | 0.1  +0.04 | -0.16  +0.06 | 1.07  +0.18 |
| 5-8 cells  MC | 0.28  +0.06 | 0.45  +0.1 | -0.08 ^g^  +0.03 | -0.1  +0.07 | -0.08  +0.08 | -1.64  +0.2 | 0.34  +0.13 | -0.07  +0.05 | -4.02  +0.54 | 2.08  +0.2 | 0.08  +0.02 | 0.26  +0.06 | 0.04  +0.01 | 0.18  +0.02 | 0.22  +0.05 | 0.09  +0.03 | -0.14  +0.07 | 0.83  +0.13 |
|  |  |  |  |  |  |  |  |  |  |  |  |  |  |  |  |  |  |  |
| Morula  NO MC | 0.25  +0.1^a^ | -0.17 ^d^  +0.23 | -0.09  +0.06 | -0.48  +0.12 ^h^ | 0.29  +0.13 | -0.1  +0.21 | 0.7  +0.11 | 0.24  +0.19 | -4.05  +0.32 | 4.61  +0.4 ^p^ | 0.26  +0.04 ^s^ | 0.2  +0.06 | 0.03  +0.02 | 0.34  +0.06 | 0.3  +0.04 | 0.14  +0.05 | 0.18  +0.08 | 1.42  +0.16 |
| Morula  MC | -0.87  +0.30 ^a^ | -3.84  +0.85 ^d^ | -0.19  +0.06 | -0.94  +0.1 ^h^ | -0.04  +0.05 | -0.48  +0.34 | 0.86  +0.07 | -0.18  +0.05 | -4.79  +0.41 | 7.61  +0.72 ^p^ | 0.13  +0.03 ^s^ | -0.38  +0.44 | 0.01  +0.02 | 0.23  +0.05 | 0.34  +0.05 | 0.11  +0.05 | 0.18  +0.05 | 1.52  +0.22 |
|  |  |  |  |  |  |  |  |  |  |  |  |  |  |  |  |  |  |  |
| Early Bl  NO MC | 1.56  +0.27 ^b^ | -4.11  +0.49 ^e^ | -0.01  +0.06 | -0.13  +0.15 ^i^ | -0.06  +0.09 | 1.94  +0.28 ^k^ | 0.51  +0.09 | 0.04  +0.06 | -2.72  +0.29 ^n^ | 7.15  +0.48 ^q^ | 0.28  +0.03 ^t^ | 0.64  +0.07 | 0.04  +0.02 ^v^ | 0.41  +0.05 | 0.30  +0.05 | -0.01  +0.05 ^y^ | 0.42  +0.11 ^aa^ | 0.4  +0.16 |
| Early Bl MC | -1.26  +0.81 ^b^ | -7.76  +0.93 ^e^ | 0.07  +0.05 | -0.63  +0.11 ^i^ | 0.03  +0.05 | 4.51  +0.74 ^k^ | 0.51  +0.07 | -0.03  +0.05 | -4.56  +0.54 ^n^ | 11.06  +0.97 ^q^ | 0.14  +0.03 ^t^ | 0.55  +0.09 | -0.08  +0.04 ^v^ | 0.25  +0.08 | 0.47  +0.11 | -0.3  +0.07 ^y^ | 0.03  +0.1 ^aa^ | 0.56  +0.2 |
|  |  |  |  |  |  |  |  |  |  |  |  |  |  |  |  |  |  |  |
| Expand Bl  NO MC | 0.69  +0.58 ^c^ | -9.28  +0.96 ^f^ | -0.01  +0.07 | -0.24  +0.18 ^j^ | -0.04  +0.06 | 5.26  +0.49 | 1.42  +0.44 ^l^ | 0.1  +0.07 ^m^ | -7.28  +0.68 ^o^ | 13.96  +0.88 ^r^ | 0.22  +0.04 ^u^ | 0.92  +0.1 | -0.14  +0.03 ^w^ | 0.47  +0.1 ^x^ | 0.73  +0.15 | -0.33  +0.06 ^z^ | 0.26  +0.15 ^bb^ | 1.00  +0.19 |
| Expand Bl  MC | -3.19  +0.92 ^c^ | -15.81  +1.48 ^f^ | -0.11  +0.07 | -0.85  +0.14 ^j^ | -0.04  +0.06 | 5.69  +0.46 | 0.41  +0.07 ^l^ | -0.14  +0.06 ^m^ | -11.57  +0.99 ^o^ | 18.18  +1.02 ^r^ | 0.04  +0.04 ^u^ | 0.82  +0.1 | -0.38  +0.04 ^w^ | 0.03  +0.11 ^x^ | 0.9  +0.17 | -0.88  +0.1 ^z^ | -0.67  +0.16 ^bb^ | 0.97  +0.16 |

Value show pmol/embryo/h and are expressed as mean + SEM. Numbers are given in the main text.

Negative values indicate amino acid consumption Positive values indicate amino acid production

Values with the same superscript are significantly different (p<0.05) NO MC = No morphological changes MC = Morphological changes
